# Preclinical data and safety assessment of phage therapy in humans

**DOI:** 10.1016/j.copbio.2021.03.002

**Published:** 2021-04

**Authors:** Janet Y Nale, Martha RJ Clokie

**Affiliations:** Department of Genetics and Genome Biology, University of Leicester, University Road, LE1 7RH, UK

## Abstract

Bacteriophages (phages) are natural biological entities that kill bacteria with species specific precision, rendering them attractive for therapeutic purposes. Phages were discovered over a century ago, but, after antibiotic discovery, their use as antimicrobials dwindled. Interest in phage therapy has, however, been rekindled by increasing multi-drug resistance to routine and frontline antibiotics and by the slowing of antibiotic innovations. To build on fundamental phage research studies and compassionate usage, information on safety and efficacy of phages is needed to motivate clinical trials and are necessary for phage therapy to become mainstream. In this review, we discussed essential phage characterisation parameters alongside the merits and limitations of state-of-the-art models to gather preclinical data on the safety and efficacy of phage therapeutics.

## Highlights

• Phages kill antibiotic resistant/sensitive bacteria so are promising therapeutics.
• Safety and efficacy phage data is needed to motivate clinical trials.
• Careful *in vitro* studies can ensure the most promising phages are developed.
• Studies on human cell lines provides key data on human-phage interactions.
• Artificial, insect and animal models can inform and direct phage development.

## Graphical abstract

**Figure:**
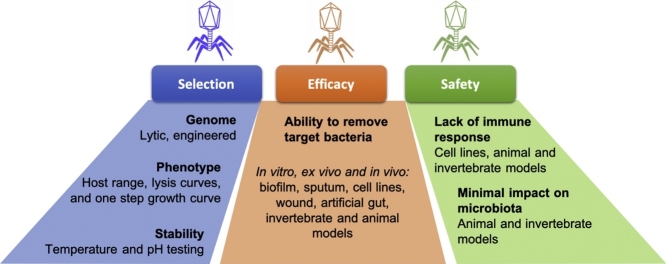

**Current Opinion in Biotechnology** 2021, **68**:310–317

This review comes from a themed issue on **Nanobiotechnology – phage therapy**

Edited by **Rob Lavigne** and **Martin J Loessner**

For a complete overview see the Issue and the Editorial

Available online 13th April 2021

**https://doi.org/10.1016/j.copbio.2021.03.002**

0958-1669/Crown Copyright © 2021 Published by Elsevier Ltd. This is an open access article under the CC BY license (http://creativecommons.org/licenses/by/4.0/).

## Introduction

Although the discovery of bacteriophages or phages (bacterial viruses) was over a century ago, there has been a recent global resurgence of interest into their therapeutic development for human bacterial infections [1,2]. This is evident from the increasing application of phage therapy in Western medicine for human compassionate use and from previous and planned clinical trials to use phages in humans [3,4]. There is also a notable increase in published studies from academic researchers and in interest from the media, regulatory agencies, patients, and clinicians who are keen to understand and use phages in human therapeutic applications [4,5].

This renewed interest in phages as precision therapeutics is based on their selective ability to target and kill specific bacterial groups but preserve commensal microbiomes [6]. Phages also replicate at infection sites, ensuring a continuous dose supply and they can penetrate difficult to treat biofilms [7]. These biological advantages seem attractive with the backdrop of continuing development of bacterial resistance to major frontline antibiotics. The associated clinical and economic burden, and the limited new antibiotic innovations are other factors motivating phage research [8,9]. Growing antimicrobial resistance (AMR) problems are worse in developing countries where antibiotic control is less stringent, and many of the projected AMR related deaths are predicted to be in Africa and Southeast Asia [8,10]. Thus, to help mitigate against AMR in such countries, increasing effort is being made from researchers in the West to encourage phage therapeutic research and development in these parts of the world [11,12].

Although obligately lytic phages (phages which strictly infect then lyse bacteria) are preferred for therapeutic use, some temperate phages (phages which have the capacity to integrate into the genome of the bacterial host) have also been demonstrated to reduce or eradicate human bacterial pathogens *in vitro* and *in vivo* [13, 14, 15]. Advances in synthetic biology and engineering have provided sophisticated platforms to engineer phages, including temperate phages to improve their clinical coverage, potency and ability to eradicate bacterial pathogens.

Engineering approaches centre on either deleting unwanted genes or by modifying or reprogramming their genomes to carry phage-delivered therapeutic biomolecules, such as CRISPR cassettes [16, 17, 18, 19]. The efficacy of these methods is being investigated to improve phage ability to control human pathogenic bacteria in various infection models [16]. Pertinent to human medicine, engineered phages have been shown to be therapeutically useful and have been incorporated into phage therapeutic cocktails. For example, in a high profile recent compassionate case phages were successfully used to remove a systemic antibiotic resistant *Mycobacterium abscessus* infection from a 15 year old teenager who had cystic fibrosis and a transplanted lung [20,21]. In this case, two of the phages used were naturally temperate but their genomes were engineered to remove the repressor gene involved in lysogeny, thus transforming them into lytic phages before being used in treatment. This case demonstrated both the successful phage treatment of this specific and pernicious pathogen and was also the first to use engineered phages in humans.

Despite the growing number of successful compassionate phage use cases, and a long history of phage use in countries such as Georgia and Russia, understanding safety concerns within existing European and global frameworks is of utmost importance to further progress phage therapy [22]. Generally, safety concerns associated with human phage therapy are linked to the reliability of phage selection and characterisation and their production under stringent regulatory and safety standards. Other concerns centre on phage pharmokinetics and pharmacodynamics and on perturbations in the microbiome due to phage activity [22, 23, 24, 25]. These worries can be largely addressed through thorough, rigorous and well-structured preclinical assays to determine safety (and efficacy) of a potential therapeutic phage product before clinical use. Such assays provide robust data on which to build a fundamental understanding of phage clinical application in humans [26].

To provide the reader with a comprehensive overview on how to develop phages, armed with our own combined backgrounds of developing phage-based products for over two decades, we reviewed recent publications on preclinical and safety data that can be collected to support the therapeutic use of phage products in human medicine. We first discuss the pre-model work needed to narrow down the choice of phages following their isolation during *in vitro* characterisation studies. We then discuss efficacy testing in the types of models most commonly used to gather preclinical data.

**Figure 1:**
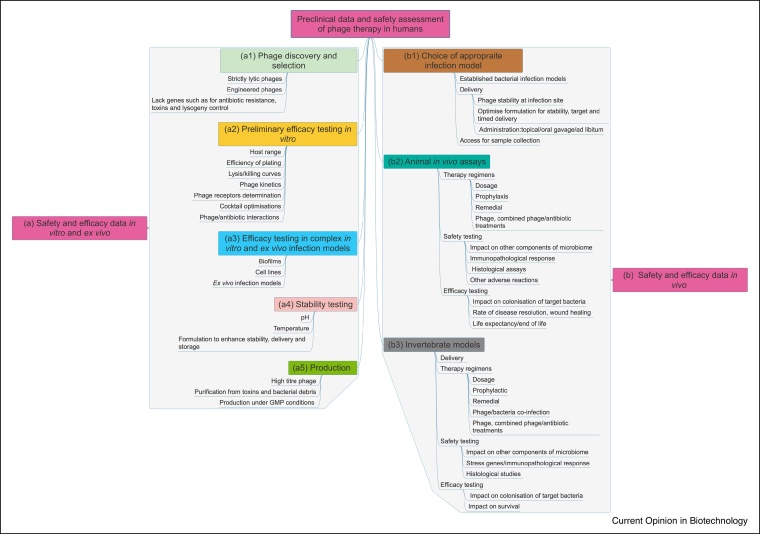
Summary of preclinical safety and efficacy data needed for human phage therapy. The boxes contain the major areas of concern and the information below summarises the relevant data that can be collected. Preclinical studies for human phage therapy should focus on initial selection of phages with suitable genomic and phenotypic properties. The phages should be naturally occurring strictly lytic or engineered phages; lacking genes that encode for antibiotic resistance or lysogeny control. They should also be stable at relevant pH and temperature conditions and produced to meet regulatory criteria. Further safety and efficacy phage characterisations *in vitro* (a, a1-5) and *in vivo* (b, b1-3) using appropriate infection models to mimic infection will provide robust preclinical data to support the translation of a potential phage product for human therapeutic use.

The pathway that depicts how phages can be moved from isolation to usage is shown in Figure 1.

## Preliminary phage discovery and *in vitro* characterisation to select phages

The fundamental principle of phage therapeutic development for clinical purposes is to ensure the potential phage product is safe and effective. The initial steps to achieve this should focus on the identification of candidate phages with suitable genomes. These should ideally be naturally occurring strictly lytic phages that do not encode genes for virulence, toxins or antimicrobial resistance, or are able to mediate horizontal gene transfer or transduce infected cells [27,28]. However, where such lytic phages are not obtainable for a bacterial species or strain(s), engineered phages can be used [21]. Phage-derived products, such as lysins and endolysins, have also been shown to have great potential for therapeutic purposes and many excellent articles have been written on the subject therefore this review does not discuss phage-based products further [29,30].

In addition to establishing the genome characteristics of specific phages, phenotypic properties are important to ascertain in order to choose phages with desirable properties. These include phage adsorption rates on relevant strains and receptors, latent period (time taken for a phage to replicate in an infected bacterium) and burst size (number of progeny phages produce per infected bacterium). All of these will provide insights into the kinetics and mechanisms by which the phages interact with the target bacterial species [26,31]. The phage lytic capability on relevant pathogenic bacterial strains is needed to determine their clinical coverage and importance for inclusion in any treatment phage combination or so called ‘phage cocktails’.

Phage host range data can be obtained through several methods to test for; including ‘spot testing’ (adding small volumes of phage suspension at a known concentration to lawns of exponentially growing culture), by determining the lysis and efficiency of phages either on Petri-dish-based assays or in microtiter assays using different multiplicities of infection (ratios of phages to bacterial cells; MOI) [14,32]. These data also provide insights into identifying suitable phage dosages [32, 33, 34]. Phages with broad host range activity that can efficiently lyse bacteria to acceptable low levels are excellent candidates for therapeutic applications [34,35]. Our data, and that from others, has consistently shown that even when efficient bacterial killing is not achievable using individual phages, multiple phages can be combined together into cocktails to expand host range coverage, lysis potential and to mitigate resistance or development of lysogenic strains [14,35,36]. We showed that a resistant (or lysogenic) strain formed by exposure to one particular phage is still susceptible to infection by the other phages within in the cocktail, leading to the observed complete *Clostridioides difficile* clearance in our study [14].

In addition to testing multiple phages in a cocktail to enhance therapeutic efficacy, combining phage therapy with antibiotics has been shown to be attractive when treating bacterial infections. A phage-resistant phenotype may subvert phage activity but confer fitness trade-offs such as reduced virulence, antibiotic sensitivity and ability to colonise. This could limit bacterial growth, and thus providing a viable means to control populations of pathogenic bacteria [37,38]. A clear example of this was demonstrated using *Pseudomonas* phage OMKO1, which uses the outer membrane porin M (OprM) of the multidrug efflux systems MexAB and MexXY as a receptor [69]. Treatment of a multidrug resistant (MDR) *P. aeruginosa* (PAO1) with phage OMKO1 alters the efflux pump mechanisms, resulting in an increased sensitivity of the phage-resistant clones to tetracycline, erythromycin, and ciprofloxacin. The robustness of this effect was also observed up to 70 generations after the bacteria was treated with the phage *in vitro* [39]. These types of data are critical in the future translation of phage therapeutics for, and possible augmentation of antibiotic therapy with phages.

Experimental *in vitro* efficacy studies are clearly useful, but should as much as possible be conducted under conditions appropriate for the targeted bacteria, infection and potential therapeutic application. Therefore, stability data of the phages at various temperatures, pH ranges, and oxygen and nutrient levels is critical for their therapeutic use [40]. When needed, phages can be formulated in pH, temperature or chemically stable forms to ensure optimal preservation and delivery to infection sites [41,42].

Future developments in phage selection can be informed by obtaining a greater understanding of what defines a ‘good phage’. A recent study based on transcriptomic profiling suggested that phage functional types could be identified based on the specific strategy that is used by a phage when it infects a bacterial host [43]. Transcriptome profiling identifies how many phage genes are expressed during the different stages of transcriptional take-over (early, middle and late), and therefore does not rely on predicted function to see if the phage expresses a lot of genes at specific stages. We do not know yet, but further study may link particularly expression profiles with efficacy. Furthermore, through extensive high-throughput phenotypic studies that link to genome studies, we will hopefully be able to identify the genomic and phenotypic attributes that phages need to be therapeutically effective *in vivo* [43]. It is also worth stating that the future application of machine learning to probe genomic features, in conjunction with hight throughput assays, is likely to significantly enhance the identification of suitable phages.

## Efficacy testing in complex *in vitro* infection models

Having established the basic infection parameters and the lysis and stability profiles of potential new phage products *in vitro*, it is essential to determine their effectiveness in the context of the infection in question *in vitro* in order to ascertain if the anticipated efficacy can be obtained. This can be achieved through phage efficacy testing in defined polymicrobial or complex bacterial communities such as biofilms, appropriate cell lines or simulated *ex vivo* infection model [44, 45, 46, 47, 48]. The most informative and useful models are discussed below with an emphasis on their merits and limitations.

### Biofilms

Human pathogenic bacteria often grow as biofilms; highly structured heterogenous aggregations of bacterial cells protected by a polymeric matrix that they exude. Immobile bacterial cells within biofilms are phenotypically and physiologically distinct from planktonic cells and have low susceptibility and penetration to antimicrobials. Antimicrobial access to molecular targets is hampered by over expression of conventional resistance mechanisms such as efflux pumps, which mediate expulsion of antibiotics out of the cells [49,50]. These resistance mechanisms complicate conventional antibiotic therapy hence, novel therapies such as phages, that penetrate biofilm are a welcome approach to treat bacterial infection [7]. In many elegant biofilm studies, both *in vitro* and *in vivo*, phages have demonstrated their efficacy to penetrate established biofilms and eradicate bacteria [44,47]. Phages encoding enzymes (such as depolymerase) which can chemically degrade the extracellular polymeric substances found in biofilms have been shown to be particularly useful for these purposes [45]. Importantly, phages are highly effective in pre-treatment studies at preventing biofilm formation of pathogenic bacteria associated with nosocomial, oral, urinary tract and wound infections [44, 45, 46, 47, 48]. Interestingly, phages have also been shown to be an excellent adjunct to antibiotics to clear *Staphylococcus aureus* in biofilm, further supporting combined phage/antibiotic therapy [51].

### Cell lines

Further phage therapy testing on appropriate cell lines to mimic infection sites provides extended information on uptake, and phage/bacterial, phage/cell lines and on bacterial/cell lines interactions. Such studies provide valuable data on immune responses, safety, efficacy and toxicity to support phage treatment of pathogenic bacteria in clinical settings [52, 53, 54]. Cell lines that have been used in phage studies include several human cerebral microvascular endothelial cells, epithelial cells most commonly HeLa, A549 and HT29 from the cervix, lung and colon, respectively [52,53,55]. Useful fibroblast cell lines used include BJ from human skin, the endothelial cell line HUVEC from umbilical veins, and monocyte-induced macrophages-THP-1 cells [55].

To illustrate the power of these studies, we discuss phage K1F that infects *Escherichia coli* EV36 displaying the K1 capsule in the presence of human cerebral microvascular endothelial cells (hCMECs) in an *in vitro* bacterial neonatal meningitis model [53]. Data from flow cytometry, confocal and live microscopy showed that the bacterium infected hCMECs in a time-dependent and concentration-dependent manner, and that the phage successfully entered the human cells, but was degraded by constitutive-dependent and Pathogen Associated Molecular Patterns (PAMP)-dependent LC3-assisted phagocytosis. The study also showed that the phage efficiently killed intracellular *E. coli* efficiently and did not induce expression of inflammatory cytokines TNFα, IL-6, IL-8 or IFNβ, and furthermore, decreased the barrier resistance of the hCMECs. This study demonstrates the potential of cell lines to generate relevant preclinical data to support translation of phage therapeutic use in neonatal meningitis. Data from the study also suggest that *in vitro* assays may actually be harder to observe effective phage behaviour in than when phages are applied in relevant settings.

### *Ex vivo* models

More complicated *ex vivo* infection models, such as batch fermentation, sputum or wound models that mimic components of the environment in which the target disease occur, are invaluable tools in which to study the ability of phages to eradicate target bacteria in a complex disease setting than offered by cell lines alone [6,56,57]. Phages have been shown not to be detrimental to the human microbiomes but can modulate it in diverse beneficial ways [58]. A particularly useful feature therefore of these models is that, in addition to studying phage efficacy, complex infection models can be used to determine potential synergistic or antagonistic impacts on the human commensal microbiome or efficacy in a mixed bacterial communities [6,57].

To discuss this approach, we highlight a recent study on *Enterococcus faecium* phage, vB_EfaS-Zip (Zip) and *Enterococcus faecalis* phage, vB_EfaP-Max (Max) in an *ex vivo* biofilm collagen wound model [57]. The model is well established, where the bacterial cells adhere to a collagen matrix, mimicking an *in vivo* wound. Biofilms of each of the organisms in this wound model were successfully treated with their appropriate phages and showed a significant reduction in bacterial numbers 3 hour post-phage treatment. However, bacterial regrowth was observed 6 hour and 8 hour post -phage treatment in the *E. faecalis* and *E. faecium* biofilms, respectively, with the development of phage resistant strains. A cocktail of both phages showed greater efficacy in dual *E. faecalis* and *E. faecium* biofilms although bacterial regrowth was still observed. In addition to efficacy, the phages were stable at various temperatures and pHs, and were not toxic to the 3T3 cells. These data support the use of phages in a wound setting. Clearly, further work is required to optimise phage cocktails or combining with antibiotics to mitigate against resistance.

### Safety and efficacy testing *in vivo*

Although *in vitro* and *ex vivo* studies provide valuable information on the potential usefulness of phages and set clear and convincing rationales for future therapeutic development, data obtained from these models are clearly not sufficient to infer safety and efficacy in complex human systems. Thus, advanced testing in relevant *in vivo* infection models is still paramount for human clinical application to proceed [26]. This is because phage testing in animals takes into account the interplay of key and complex factors in human physiological and immune systems, which cannot be fully investigated *in vitro* or *ex vivo*. Animal models have been studied for several bacterial infections where infections were induced through antibiotic treatment and animals have display diseased symptoms similar to humans. Thus, information on phage safety, efficacy, toxicology and pharmacokinetics from these models is needed to translate phages to human clinical use [25,59,60].

### Animal phage therapy studies

For effective evaluation of the activity of therapeutic phages in animals as well as other infection models, it is important to ensure that highly purified preparations are used to exclude potential contaminants that can interfere with activity [61]. High titre phage preparations should be free from endotoxins, exotoxins and other bacterial cell debris contaminations arising from phage lysis of bacteria. These impurities can be removed using procedures such as multiple low-speed centrifugations, microfiltration, cross-flow ultrafiltration, anion exchange chromatography, octanol extraction and endotoxin removal columns [62,63].

Having established the purity of the phage preparation, it is vital to determine the best delivery method in order to reflect how the phages will ultimately be given to humans. This depends on the disease model, the intended target indication, and on the stability of the phages. Where stability is a limitation, as mentioned above, phages can be formulated for improved stability and programmed delivery [41,42]. Important parameters to guide phage testing in animals include: the therapeutic regimen (whether prophylaxis or remedial) to be tested, the length of experiments, phage dosage, assessment of vital signs and symptoms to determine scheduled end-point, sampling to determine impact on bacterial colonisation, histopathological and immunological studies or wound healing [14,64, 65, 66].

The choice of animal model also depends on the disease and the way it mimics human infection in the specific animal, and consequently several animals have been studied for phage therapy applications [59]. To illustrate how animal models advance therapeutic development, we refer to the study designed to investigate phage efficacy against an MDR *Staphylococcus aureus* in a diabetic mouse wound infection model [66]. In this study, a three-phage cocktail was produced under current good manufacturing practice conditions. Excisional wounds were inflicted on diabetic induced female Balb/c mice and infected with MDR *S. aureus* SA63-2498 test strain before treatment with the phage cocktail. The phage-treated *S. aureus*-colonised mice groups were compared to those treated with vancomycin and to a saline-only control. The data showed a decrease in bacterial load and wound size in the phage-treated and antibiotic-treated mice groups. In contrast, wounds in the control *S. aureus*-colonised mice increased and became inflamed, ulcerated and suppurating. In summary, the data demonstrated that phage treatment is comparable to vancomycin in efficacy, thus supporting translation of the phages for clinical application in diabetic wounds [66].

### Phage efficacy testing in refined *in vivo* models

Despite the importance of animal models to phage therapy studies, there are inherent technical limitations associated with expertise, the long process of licence and ethics approvals and costs of animal husbandry. To circumvent these limitations on animal use in phage preclinical studies, and to provide translation data to guide animal usage, invertebrate models have been successfully developed and are increasingly used [59]. These models are inexpensive so facilitate extensive replicates and testing of different treatment regimens. They also require less expertise to work with, and their cells are similar enough to human cells to allow comparable impact assessment and inference [44]. Phages have been shown to be stable in these models and the route of phage administration depends on the pathogen under investigation [44,59,67]. Through colonisation, survival and immune response studies, these models have been instrumental in determining phage therapeutic potential [44,59,67]. In a recent phage application study, carbapenem-resistant *Acinetobacter baumannii* infected *Galleria mellonella* larvae were treated with phage Bφ-R2096 via the proleg [68^•^]. The survival of phage treated larvae was 50% at 24 hour post treatment compared to 0% in the control group. Histological studies also showed less tissue damage and melanisation in the fat body wall and muscle layer in the phage treated group compared to the controls [68^•^]. Similarly, our work showed that phages could prevent *C. difficile* colonisation in insects when an optimised phage cocktail was administered into the larvae via oral gavage and that multiple doses of phages could be used to successfully remove bacterial colonisation and prolong the life of the larvae [44,67].

## Conclusion

The rise in multi-drug resistance in hospitals and the associated clinical and economic detrimental impacts motivates the urgent need for innovative anti-infectives to supplement or replace classical antibiotics. Phages have target specificity and can kill antibiotic resistant strains, and, as such, are viable alternatives to antibiotics. We have described several models by which phages have been shown to prevent and control the growth of human pathogenic bacteria. Such studies will be incredibly valuable to provide the preclinical safety and efficacy data needed to translate the potential of phages to a human setting. We analysed several relevant recent literature in the field and discussed the specific merits of the models to produce robust preclinical data, focusing on aspects relating to phage selection and analyses *in vitro*, *ex vivo* and *in vivo.* Data from biologically relevant models will inform translation of potential therapeutic phage products to human clinical use.

## Conflict of interest statement

Nothing declared.

## References and recommended reading

Papers of particular interest, published within the period of review, have been highlighted as:

• of special interest

## References

[1] Aslam S., Schooley R.T. (2019). What’s old is new again: bacteriophage therapy in the 21st century. Antimicrob Agents Chemother.

[2] Hesse S., Adhya S. (2019). Phage therapy in the twenty-first century: facing the decline of the antibiotic era; is it finally time for the age of the phage?. Annu Rev Microbiol.

[3] Aslam S., Lampley E., Wooten D., Karris M., Benson C., Strathdee S., Schooley R.T. (2020). Lessons learned from the first 10 consecutive cases of intravenous bacteriophage therapy to treat multidrug-resistant bacterial infections at a single center in the United States. Open Forum Infect Dis.

[4] Górski A., Borysowski J., Międzybrodzki R. (2020). Phage therapy: towards a successful clinical trial. Antibiotics.

[5] Żaczek M., Weber-Dąbrowska B., Międzybrodzki R., Łusiak-Szelachowska M., Górski A. (2020). Phage therapy in Poland – a centennial journey to the first ethically approved treatment facility in Europe. Front Microbiol.

[6] Nale J., Redgwell T.A., Millard A., Clokie M.R.J. (2018). Efficacy of an optimised bacteriophage cocktail to clear *Clostridium difficile* in a batch fermentation model. Antibiotics.

[7] Melo L.D.R., Pires D.P., Monteiro R., Azeredo J., Górski A., Międzybrodzki R., Borysowski J. (2019). Phage therapy of infectious biofilms: challenges and strategies. Phage Therapy: A Practical Approach.

[8] O’Neill J. (2016). Tackling Drug-resistant Infections Globally: Final Report and Recommendations.

[9] Brives C., Pourraz J. (2020). Phage therapy as a potential solution in the fight against AMR: obstacles and possible futures. Palgrave Commun.

[10] Pokharel S., Raut S., Adhikari B. (2019). Tackling antimicrobial resistance in low-income and middle-income countries. BMJ Glob Health.

[11] Nagel T.E., Chan B.K., Nale J.Y., Clokie M.R.J., Carlet J., Upham G. (2018). Phages as antibacterial agents: laboratory training in developing countries. AMR Control 2018 Overcoming Global Antimicrobial Resistance.

[12] Nagel T.E., Chan B.K., De Vos D., El-Shibiny A., Kang’ethe E.K., Makumi A., Pirnay J.-P. (2016). The developing world urgently needs phages to combat pathogenic bacteria. Front Microbiol.

[13] Furfaro L.L., Payne M.S., Chang B.J. (2018). Bacteriophage therapy: clinical trials and regulatory hurdles. Front Cell Infect Microbiol.

[14] Nale J.Y., Spencer J., Hargreaves K.R., Buckley A.M., Trzepiński P., Douce G.R., Clokie M.R.J. (2016). Bacteriophage combinations significantly reduce *Clostridium difficile* growth in vitro and proliferation in vivo. Antimicrob Agents Chemother.

[15] Monteiro R., Pires D.P., Costa A.R., Azeredo J. (2019). Phage therapy: going temperate?. Trends Microbiol.

[16] Selle K., Fletcher J.R., Tuson H., Schmitt D.S., McMillan L., Vridhambal G.S., Rivera A.J., Montgomery S.A., Fortier L.C., Barrangou R. (2020). In vivo targeting of *Clostridioides difficile* using phage-delivered CRISPR-Cas3 antimicrobials. mBio.

[17] Kilcher S., Studer P., Muessner C., Klumpp J., Loessner M.J. (2018). Cross-genus rebooting of custom-made, synthetic bacteriophage genomes in L-form bacteria. Proc Natl Acad Sci U S A.

[18] Brown R., Lengeling A., Wang B. (2017). Phage engineering: how advances in molecular biology and synthetic biology are being utilized to enhance the therapeutic potential of bacteriophages. Quant Biol.

[19] Yehl K., Lemire S., Yang A.C., Ando H., Mimee M., Torres M.D.T., de la Fuente-Nunez C., Lu T.K. (2019). Engineering phage host-range and suppressing bacterial resistance through phage tail fiber mutagenesis. Cell.

[20] Mayor S. (2019). Sixty seconds on. . . bacteriophages. BMJ.

[21] Dedrick R.M., Guerrero-Bustamante C.A., Garlena R.A., Russell D.A., Ford K., Harris K., Gilmour K.C., Soothill J., Jacobs-Sera D., Schooley R.T. (2019). Engineered bacteriophages for treatment of a patient with a disseminated drug-resistant *Mycobacterium abscessus*. Nat Med.

[22] Nair A., Khairnar K. (2019). Genetically engineered phages for therapeutics: proceed with caution. Nat Med.

[23] Kincaid R., Górski A., Międzybrodzki R., Borysowski J. (2019). Treatment and prevention of bacterial infections using bacteriophages: perspectives on the renewed interest in the United States. Phage Therapy: A Practical Approach.

[24] Principi N., Silvestri E., Esposito S. (2019). Advantages and limitations of bacteriophages for the treatment of bacterial infections. Front Pharmacol.

[25] Nilsson A.S. (2019). Pharmacological limitations of phage therapy. Upsala J Med Sci.

[26] Abedon S.T. (2017). Information phage therapy research should report. Pharmaceuticals (Basel).

[27] Fernández L., Gutiérrez D., García P., Rodríguez A. (2019). The perfect bacteriophage for therapeutic applications-a quick guide. Antibiotics (Basel, Switzerland).

[28] Hyman P. (2019). Phages for phage therapy: isolation, characterization, and host range breadth. Pharmaceuticals (Basel, Switzerland).

[29] Villa G.T., Sieiro C. (2020). Phage therapy, lysin therapy, and antibiotics: a trio due to come. Antibiotics (Basel, Switzerland).

[30] Kropinski A.M. (2018). Bacteriophage research - what we have learnt and what still needs to be addressed. Res Microbiol.

[31] Thanki A.M., Taylor-Joyce G., Dowah A., Nale J.Y., Malik D.J., Clokie M.R.J. (2018). Unravelling the links between phage adsorption and successful infection in *Clostridium difficile*. Viruses.

[32] Phothichaisri W., Ounjai P., Phetruen T., Janvilisri T., Khunrae P., Singhakaew S., Wangroongsarb P., Chankhahaengdecha S. (2018). Characterization of bacteriophages infecting clinical isolates of *Clostridium difficile*. Front Microbiol.

[33] Sergueev K.V., Filippov A.A., Farlow J., Su W., Kvachadze L., Balarjishvili N., Kutateladze M., Nikolich M.P. (2019). Correlation of host range expansion of therapeutic bacteriophage Sb-1 with allele state at a hypervariable repeat locus. Appl Environ Microbiol.

[34] Manohar P., Tamhankar A.J., Lundborg C.S., Nachimuthu R. (2019). Therapeutic characterization and efficacy of bacteriophage cocktails infecting *Escherichia coli*, *Klebsiella pneumoniae*, and *Enterobacter* species. Front Microbiol.

[35] Jasim H.N., Hafidh R.R., Abdulamir A.S. (2018). Formation of therapeutic phage cocktail and endolysin to highly multi-drug resistant *Acinetobacter baumannii*: in vitro and in vivo study. Iran J Basic Med Sci.

[36] Yuan Y., Wang L., Li X., Tan D., Cong C., Xu Y. (2019). Efficacy of a phage cocktail in controlling phage resistance development in multidrug resistant *Acinetobacter baumannii*. Virus Res.

[37] Mangalea M.R., Duerkop B.A. (2020). Fitness trade-offs resulting from bacteriophage resistance potentiate synergistic antibacterial strategies. Infect Immun.

[38] Burmeister A.R., Fortier A., Roush C., Lessing A.J., Bender R.G., Barahman R., Grant R., Chan B.K., Turner P.E. (2020). Pleiotropy complicates a trade-off between phage resistance and antibiotic resistance. Proc Natl Acad Sci U S A.

[39] Gurney J., Pradier L., Griffin J.S., Gougat-Barbera C., Chan B.K., Turner P.E., Kaltz O., Hochberg M.E. (2020). Phage steering of antibiotic-resistance evolution in the bacterial pathogen, *Pseudomonas aeruginosa*. Evol Med Public Health.

[40] Jończyk-Matysiak E., Łodej N., Kula D., Owczarek B., Orwat F., Międzybrodzki R., Neuberg J., Bagińska N., Weber-Dąbrowska B., Górski A. (2019). Factors determining phage stability/activity: challenges in practical phage application. Expert Rev Anti Infect Ther.

[41] Vinner G.K., Vladisavljević G.T., Clokie M.R.J., Malik D.J. (2017). Microencapsulation of *Clostridium difficile* specific bacteriophages using microfluidic glass capillary devices for colon delivery using pH triggered release. PLoS One.

[42] Malik D.J., Sokolov I.J., Vinner G.K., Mancuso F., Cinquerrui S., Vladisavljevic G.T., Clokie M.R.J., Garton N.J., Stapley A.G.F., Kirpichnikova A. (2017). Formulation, stabilisation and encapsulation of bacteriophage for phage therapy. Adv Colloid Interface Sci.

[43] Clokie M.R.J., Blasdel B.G., Demars B.O.L., Sicheritz-Pontén T. (2020). Rethinking phage ecology by rooting it within an established plant framework. Phage.

[44] Nale J.Y., Chutia M., Carr P., Hickenbotham P., Clokie M.R.J. (2016). ‘Get in early’; biofilm and wax moth (*Galleria mellonella*) models reveal new insights into the therapeutic potential of *Clostridium difficile* bacteriophages. Front Microbiol.

[45] Ferriol-González C., Domingo-Calap P. (2020). Phages for biofilm removal. Antibiotics (Basel).

[46] Chegini Z., Khoshbayan A., Taati Moghadam M., Farahani I., Jazireian P., Shariati A. (2020). Bacteriophage therapy against *Pseudomonas aeruginosa* biofilms: a review. Ann Clin Microbiol Antimicrob.

[47] Doub J.B. (2020). Bacteriophage therapy for clinical biofilm infections: parameters that influence treatment protocols and current treatment approaches. Antibiotics (Basel, Switzerland).

[48] Huon J.-F., Montassier E., Leroy A.-G., Grégoire M., Vibet M.-A., Caillon J., Boutoille D., Navas D. (2020). Phages versus antibiotics to treat infected diabetic wounds in a mouse model: a microbiological and microbiotic evaluation. mSystems.

[49] Zaborskyte G., Andersen J.B., Kragh K.N., Ciofu O. (2017). Real-time monitoring of nfxB mutant occurrence and dynamics in *Pseudomonas aeruginosa* biofilm exposed to subinhibitory concentrations of ciprofloxacin. Antimicrob Agents Chemother.

[50] Crabbé A., Jensen P.Ø, Bjarnsholt T., Coenye T. (2019). Antimicrobial tolerance and metabolic adaptations in microbial biofilms. Trends Microbiol.

[51] Dickey J., Perrot V. (2019). Adjunct phage treatment enhances the effectiveness of low antibiotic concentration against *Staphylococcus aureus* biofilms in vitro. PLoS One.

[52] Shan J., Ramachandran A., Thanki A.M., Vukusic F.B.I., Barylski J., Clokie M.R.J. (2018). Bacteriophages are more virulent to bacteria with human cells than they are in bacterial culture; insights from HT-29 cells. Sci Rep.

[53] Møller-Olsen C., Ross T., Leppard K.N., Foisor V., Smith C., Grammatopoulos D.K., Sagona A.P. (2020). Bacteriophage K1F targets *Escherichia coli* K1 in cerebral endothelial cells and influences the barrier function. Sci Rep.

[54] Huh H., Wong S., St. Jean J., Slavcev R. (2019). Bacteriophage interactions with mammalian tissue: therapeutic applications. Adv Drug Deliv Rev.

[55] Bichet M.C., Chin W.H., Richards W., Lin Y.-W., Avellaneda-Franco L., Hernandez C.A., Oddo A., Chernyavskiy O., Hilsenstein V., Neild A., Li J., Hans Voelcker N., Patwa R., Barr J.J. (2021). Bacteriophage uptake by mammalian cell layers represents a potential sink that may impact phage therapy. iScience.

[56] Darch S.E., Kragh K.N., Abbott E.A., Bjarnsholt T., Bull J.J., Whiteley M. (2017). Phage inhibit pathogen dissemination by targeting bacterial migrants in a chronic infection model. mBio.

[57] Melo L.D.R., Ferreira R., Costa A.R., Oliveira H., Azeredo J. (2019). Efficacy and safety assessment of two enterococci phages in an in vitro biofilm wound model. Sci Rep.

[58] Divya Ganeshan S., Hosseinidoust Z. (2019). Phage therapy with a focus on the human microbiota. Antibiotics (Basel, Switzerland).

[59] Brix A., Cafora M., Aureli M., Pistocchi A. (2020). Animal models to translate phage therapy to human medicine. Int J Mol Sci.

[60] Kaabi S.A.G., Musafer H.K. (2019). An experimental mouse model for phage therapy of bacterial pathogens causing bacteremia. Microb Pathog.

[61] Luong T., Salabarria A., Roach D.R. (2020). Phage therapy in the resistance era: where do we stand and where are we going?. Clin Ther.

[62] Luong T., Salabarria A., Edwards R.A., Roach D.R. (2020). Standardized bacteriophage purification for personalized phage therapy. Nat Protoc.

[63] Hietala V., Horsma-Heikkinen J., Carron A., Skurnik M., Kiljunen S. (2019). The removal of endo- and enterotoxins from bacteriophage preparations. Front Microbiol.

[64] Abd El-Aziz A.M., Elgaml A., Ali Y.M. (2019). Bacteriophage therapy increases complement-mediated lysis of bacteria and enhances bacterial clearance after acute lung infection with multidrug-resistant *Pseudomonas aeruginosa*. J Infect Dis.

[65] Anand T., Virmani N., Kumar S., Mohanty A.K., Pavulraj S., Bera B.C., Vaid R.K., Ahlawat U., Tripathi B.N. (2020). Phage therapy for treatment of virulent *Klebsiella pneumoniae* infection in a mouse model. J Global Antimicrob Resist.

[66] Kifelew L.G., Warner M.S., Morales S., Vaughan L., Woodman R., Fitridge R., Mitchell J.G., Speck P. (2020). Efficacy of phage cocktail AB-SA01 therapy in diabetic mouse wound infections caused by multidrug-resistant *Staphylococcus aureus*. BMC Microbiol.

[67] Nale J.Y., Chutia M., Cheng J.K.J., Clokie M.R.J. (2020). Refining the *Galleria mellonella* model by using stress marker genes to assess *Clostridioides difficile* infection and recuperation during phage therapy. Microorganisms.

[68•] Jeon J., Park J.-H., Yong D. (2019). Efficacy of bacteriophage treatment against carbapenem-resistant *Acinetobacter baumannii* in *Galleria mellonella* larvae and a mouse model of acute pneumonia. BMC Microbiol.

[69] Chan B.K., Sistrom M., Wertz J.E., Kortright K.E., Narayan D., Turner P.E. (2016). Phage selection restores antibiotic sensitivity in MDR *Pseudomonas aeruginosa*. Sci Rep.

